# Cost effectiveness of quadrivalent influenza vaccines in the elderly population of Malaysia

**DOI:** 10.1038/s41598-023-46079-y

**Published:** 2023-10-31

**Authors:** Syed Mohamed Aljunid, Nur Syazana Mad Tahir, Aniza Ismail, Aznida Firzah Abdul Aziz, Amirah Azzeri, S. A. Zafirah, Azimatun Noor Aizuddin

**Affiliations:** 1 Department of Public Health and Community Medicine, School of Medicine, International Medical University, Bukit Jalil, Kuala Lumpur, Malaysia; 2 International Centre for Casemix and Clinical Coding, Hospital Canselor Tuanku Muhriz, Universiti Kebangsaan Malaysia, Cheras, Kuala Lumpur Malaysia; 3 Department of Public Health Medicine, Faculty of Medicine, Universiti Kebangsaan Malaysia, Cheras, Kuala Lumpur Malaysia; 4 Federal Government Administrative Centre, Ministry of Health Malaysia, Putrajaya, Malaysia; 5 Department of Family Medicine, Faculty of Medicine, Universiti Kebangsaan Malaysia, Kuala Lumpur, Malaysia; 6 Public Health Unit, Department of Primary Health Care, Faculty of Medicine and Health Sciences, Universiti Sains Islam Malaysia, Nilai, Malaysia; 7 Faculty of Medicine and Defence Health, National Defence University of Malaysia, Kuala Lumpur, Malaysia

**Keywords:** Health care economics, Health policy

## Abstract

The economic burden of influenza is a significant issue within healthcare system, related to higher medical costs particularly among the elderly. Yet, influenza vaccination rates in the elderly in Malaysia were considerably low as it is not part of Malaysia’s national immunization program, with substantial mortality and morbidity consequences. Therefore, we conducted a cost-effectiveness analysis of quadrivalent influenza vaccine (QIV) for the elderly in Malaysia compared with the current no-vaccination policy. A static cost-utility model, with a lifetime horizon based on age, was used for the analysis to assess the cost-effectiveness and health outcomes associated with QIV. Univariate and probabilistic sensitivity analyses were performed to test the effects of variations in the parameters. The use of QIV in Malaysia’s elderly population would prevent 66,326 potential influenza cases and 888 potential deaths among the elderly, leading to 10,048 potential quality-adjusted life years (QALYs) gained. The QIV would also save over USD 4.4 million currently spent on influenza-related hospitalizations and reduce productivity losses by approximately USD 21.6 million. The ICER per QALY gained from a third-party payer’s perspective would be USD 2216, which is lower than the country’s gross domestic product per capita. A QIV-based vaccination program in the elderly was found to be highly cost-effective, therefore would reduce the financial burden of managing influenza and reduce pre-mature death related to this disease.

## Introduction

Influenza is a highly infectious and acute febrile illness that primarily affects the respiratory tract. It is characterized by a sudden onset of fever (38–40 °C), cough (usually dry), headaches, myalgia, sore throat and inflammation of the respiratory tract. Severe influenza could cause complications such as pneumonia, septicemia, meningitis and cardiac complications^1^. Early screening of influenza may help lessen the symptoms and disease severity. Currently, a comprehensive action plan was developed that would allow an effective influenza management^2^. Global estimates associate influenza with 3–5 million severe cases and 290,000–650,000 respiratory deaths annually^3^. Tropical regions such as Malaysia have no clear seasonal patterns and influenza circulation is year-round, typically with several peaks during the monsoon seasons^4^. Very few studies were conducted on the burden of influenza disease in Malaysia. From 2005 to 2009, 14.0% of 7117 respiratory specimens from patients with influenza-like illness were positive for influenza virus^5^. Following this, current surveillance has reported that the influenza-positive rate ranged from 3.6 to 7.3% from the number of samples tested annually, ranging from 5391 to 9405^6^. A previous study has suggested the occurrence of influenza in Malaysia is underestimated due to the method of influenza detection^4^. Furthermore, influenza is not classified as a notifiable disease, resulting in an underrepresentation of actual cases, as a significant proportion of cases are not confirmed by laboratory testing.

Vaccination is the best strategy to prevent and control influenza and its complications^7^. Influenza vaccination is strongly recommended for those at higher risk for influenza complications such as the elderly. Prior studies have demonstrated that influenza vaccination could mitigate the risk of influenza-associated complications, reduce mortality rates among children, and protect pregnant women and people with chronic health conditions^1,8–10^. Influenza vaccination also plays a crucial role in reducing the severity of illness in infected patients^10^. The most widely available influenza vaccines are the quadrivalent influenza vaccines (QIVs), which contain two influenza A strains (H1N1 and H3N2 subtypes) and two influenza B strains (Victoria and Yamagata lineages), as per World Health Organization (WHO) recommendations. Currently, there are a few licensed vaccines available and approved to be used in elderly, which are trivalent (TIV) or quadrivalent (QIV), cell-cultured or egg-based, standard-dose or high-dose, and adjuvanted or nonadjuvanted vaccine. Based on the review of available studies, the high-dose quadrivalent influenza vaccine (QIV) is highly recommended for older people in the 2022–2023 season because it is more effective compared to other types of vaccines^11^. Besides, a consensus has been made by the Malaysian Influenza Working Group (MIWG) for the elderly to receive influenza vaccine annually^12^. However, in the event that no high-dose vaccine options are accessible, it is advisable to provide any age-appropriate influenza vaccine^12^.

The target of influenza vaccine coverage has been agreed to by countries in a World Health Assembly resolution on 28 May 2003^13^. However, a report from one tertiary care centre in Malaysia detected poor uptake of influenza vaccination among older patients^14^. Meanwhile, a nationwide study reported only 5.5% influenza vaccination coverage rates for the elderly^15^. One possible reason for the trend was that influenza vaccines are only funded for healthcare workers although healthcare services are meant to be free for the elderly in public facilities. Due to their lower immunity, the elderly are more prone to contracting influenza compared with other age groups^16^, and are particularly vulnerable to related complications. An Italian study reported that most influenza-related deaths occurred in the elderly^17^. Therefore, there is a need to include the elderly in a national-level influenza vaccination program to enhance vaccine uptake within this group.

The vaccination initiative requires a thorough consideration of potential vaccine costs and savings on treatment and outbreak control related expenses. Cost-effectiveness analyses (CEAs) are widely used to help decision makers choose the most appropriate interventions based on costs and effectiveness within a constrained budget. Across the Asia–Pacific region, several cost-effectiveness studies have been conducted in the use of influenza vaccine in the elderly^18–20^. For instance, a Korean study reported that the inclusion of influenza vaccines in the National Immunization Program (NIP) for those aged 50–64 years was cost effective, with QIVs emerging as the preferred option based on its greater protection against influenza B^21^. To the best of our knowledge, no cost-effectiveness analysis of the influenza vaccine has been performed in Malaysia. To address this gap, we used a cost-utility model to predict the public health impact and cost-effectiveness of QIVs compared with the current no-vaccination policy for individuals aged 60 years and older in Malaysia.

## Methods

### Cost of influenza

Influenza-related costs were estimated from a societal perspective, i.e. costs to the health care system, costs of over-the-counter (OTC) medication for Malaysians aged 60 years and above, and costs related to productivity losses in the working elderly and their caregivers. Influenza-related hospitalization costs were based on the Casemix database in a Malaysian teaching hospital from 2010 to 2020. Casemix is a patient classification system that groups patient treatment episodes into categories that has similar resource use and same clinical features. Casemix is also known as Diagnosis Related Groups (DRG). The Casemix system used in this study, the MY-DRG, incorporated data from clinical coding based on the International Classification of Disease 10th Revision (ICD-10) for diagnosis and International Classification of Disease, Clinical Modification 9th Revision (ICD-9-CM) for procedures^22^. All cases of influenza that were included in this study were identified according to a principal or secondary diagnosis coded in ICD-10 as J09, J10.0, J10.1, J10.8, J11.0, J11.1, J11.8, J12.8 and J12.9, representing influenza and its complications. The vaccine cost applied in this analysis was a public market price published by Sanofi Pasteur. Data from a previous local study was referenced for the administrative costs of a vaccine program^23^ and medication costs at a general practice (Table 1). The medication costs were calculated using the proportion of drug cost (27.7%) from the total provider’s cost for general practice in Malaysia^24^. Although, this was not specific for influenza treatment, the utilization in this model was agreed upon by the public health experts and it is appropriate for the Malaysia health system. Expert input and local data were gathered to estimate the cost of general practice (GP) visits and OTC medication for the treatment of influenza, respectively. Cost of GP visits was assumed to be the same as a local study done previously^24^, considering the majority of influenza-like-illness patients would visit public primary care facilities and the group of experts in the study agreed on two visits per episode of care^2^.

**Table 1:** Model input value.

Model input	Baseline value	DSA range	PSA		Sources
			Distribution type	Parameters	
Population size					
60–74 years	2,755,900	–	–	–	^37^
75 years and above	744,800	–	–	–	^37^
Life expectancy					
60–74 years	20.60	–	–	–	^38,39^
75 years and above	9.82	–	–	–	^38,39^
Discount rate, %	3	0–5	–	–	^40^
Utility parameters					
Utility norm population					
60–74 years	0.8600	–	Beta (μ,σ)	(0.972–0.100)	^29^
75 + years	0.8600	–	Beta (μ,σ)	(0.801–0.128)	^29^
QALYs loss	0.0068	0.0054–0.0082	Beta (μ,σ)	(0.015–0.001)	^30^
Vaccination coverage	75	60–48	Beta (μ,σ)	(50.0–0.1)	^13^
Non-consulting cases	0.41	0.33–0.49	Beta (μ,σ)	(0.710–0.142)	^31^
Costs (USD)					
Vaccine cost	7.14	5.71–8.57	LogNormalX (μ,σ)	(7.14–1.41)	Sanofi Pasteur
Vaccine administration	3.29	2.63–3.95	LogNormalX (μ,σ)	(3.29–0.66)	^23^
GP visit	7.05	5.64–8.46	LogNormalX (μ,σ)	(7.05–1.41)	Expert input^24^
Hospitalization	1397	1118–1677	LogNormalX (μ,σ)	(1,397–279.5)	Primary data collection (Casemix UKMMC)
Prescribe medicine (GP)	6.81	5.45–8.17	LogNormalX (μ,σ)	(6.81–1.36)	^24^
OTC medicine	37.74	30.19–45.29	LogNormalX (μ,σ)	(37.74–7.55)	Primary data collection
Productivity losses due to infection					
Workdays lost	2	1.60–2.40	LogNormalX (μ,σ)	(2.0–0.4)	Expert input^2^
Percentage of cases attributable to the B-strain, %					
2012–2013	18	14.4–21.5	Beta (μ,σ)	(17.9–3.6)	^34^
2013–2014	39	31.2–46.8	Beta (μ,σ)	(39.0–7.8)	^34^
2014–2015	27	21.3–31.9	Beta (μ,σ)	(26.6–5.3)	^34^
2015–2016	18	14.7–22.0	Beta (μ,σ)	(18.3–3.7)	^34^
2016–2017	72	58.0–87.0	Beta (μ,σ)	(72.5–14.5)	^34^
2017–2018	32	25.6–38.4	Beta (μ,σ)	(32.0–6.40)	^34^
2018–2019	41	32.8–49.2	Beta (μ,σ)	(41.00–8.20)	^34^
Vaccine efficacy					
Against A-strain					
60–74 years	59	41–71	Beta (μ,σ)	(59.0–0.09)	^36^
75 + years	59	47–70	Beta (μ,σ)	(61.0–0.07)	^36^
Against matched B-strain					
60–74 years	71	12–94	Beta (μ,σ)	(66.0–0.25)	^36^
75 + years	70	17–94	Beta (μ,σ)	(77.0–0.24)	^36^
Excess death rates per 100,000					
60–74 years	37.9	± 20%	LogNormalX (μ,σ)	(1.00–0.10)	^32^
75 + years	111.9	± 20%	LogNormalX (μ,σ)	(1.00–0.10)	^32^
Excess hospitalization rates per 100,000					
60–74 years	136.0	± 20%	LogNormalX (μ,σ)	(1.00–0.10)	^32,33^
75 + years	403.0	± 20%	LogNormalX (μ,σ)	(1.00–0.10)	^32,33^
Excess GP consultation rates per 100,000					
60–74 years	2008	± 20%	LogNormalX (μ,σ)	(1.00–0.10)	^32,33^
75 + years	5930	± 20%	LogNormalX (μ,σ)	(1.00–0.10)	^32,33^

*GP* general practitioner, *DSA* deterministic sensitivity analysis, *OTC* over-the-counter, *PSA* probabilistic sensitivity analysis, *QALY* quality-adjusted life years.

Indirect costs, which were productivity loss associated with the number of workdays lost and the average length of a hospital stay, were calculated based on expert opinion^2^ and estimates from the Casemix database. The cost was calculated using the human capital approach as previously described^25^. The estimated average daily productivity (USD 39.72) was calculated based on Malaysia’s gross domestic product (GDP) per capita in 2020^26^, based on the assumption of 40 working hours per week or 8 working hours per day. Employment rates among the elderly were referenced from a recent study done in Malaysia^27^ while rates among their caregivers were based on the Department of Statistics Malaysia reports^28^. We estimated the costs of lost productivity of their caregivers due to hospitalization and disability in this population. This is because the caregivers would need to take leave from their work to accompany their hospitalized parents. The productivity lost for the patients and caregivers reflected the societal cost in this study.

### Influenza-related health parameters

The population utility rate was based on the estimated EQ-VAS score of the general adult Malaysian population using EQ-5D^29^. Quality-adjusted life years (QALYs) lost due to influenza were based on surveillance done in the UK for influenza-like illness among people aged over 65 years^30^. The QALY loss was assumed to be the same for both age groups in the model.

The number of non-consulting influenza cases for one consultation was derived from the estimated number of patients with influenza-like illness who sought medical care in Thailand^31^. The influenza vaccination coverage rate was assumed at 75%, reflecting the 2003 World Health Assembly’s goal for the elderly^13^.

Mortality rates per 100,000 were based on published estimates from a recent study of influenza-associated mortality in Malaysians aged 60–74 years and 75 years and above^32^. Influenza-related hospitalizations rates were estimated based on the probability of hospitalization over flu infection rates^33^ for people over 65 years old in the US and applied to the mortality rates in Malaysia^32^. The same formula was used to calculate the GP consultation rates, whereby the probability of outpatient visits for influenza^33^ in non-high risk populations was applied to mortality rates from a previous published study^32^.

The proportion of illness due to influenza A, A (H1N1), A(H3N2), B, B/Yamagata, and B/Victoria for each year between 2013 until 2019 was gathered from the FluNet database for Malaysia^34^.

### Influenza vaccine effectiveness (IVE)

The average vaccine effectiveness of QIV against influenza A and influenza B, respectively were based on the method adopted in a Canadian model^16^ and an Italian study^35^. In this study, the IVE of QIV against influenza A and matched B-lineage influenza were derived from Clement et al.^36^ and adjusted according to the age groups included in the model. The same source was referenced to estimate IVE against mismatched-influenza B by using the cross-protection rates from the study^36^. The assumption was that IVE against influenza A(H1N1) and A(H3N2) would be identical. IVE against health outcomes like influenza-related GP visits, hospitalizations, and death in each age group were calculated as described for the Canadian^16^ and Italian^35^ model.

### Model structure and input

Our modelling strategy was to estimate the incremental cost-effectiveness ratio (ICER) among the Malaysian elderly population who were vaccinated with QIVs versus those who were not. We developed a static cost-utility model to determine the health and economic impact, as previously designed in Canada^16^ and Italy^35^, to estimate the cost-effectiveness of QIVs. The previous published model has described the impact of switching TIV to QIV with three basic simulations including no vaccination, TIV and QIV and estimated the outcomes of each strategy among all aged groups. Their parameters input was selected based on their country’s preference and data availability. Both of these studies evaluate the cost-effectiveness from the societal and provider perspective^16,35^. In contrast, our model described the cost-effectiveness and health outcome of QIV and no vaccination strategy in the elderly mainly from the third-party payer perspective. Health outcomes prevented with QIVs were estimated by subtracting the expected rates of influenza-related health outcomes with QIVs from observed rates in non-vaccinated populations. The outputs included were health-related benefits and the number of QALYs gained, life-years gained and saved workdays. Influenza cases prevented were estimated by adding non-consulting cases to GP visits. The elderly population in the model were stratified into two groups: those aged 60 to 74 years and 75 years and above. The costs of each outcome were derived by multiplying the outcome by the estimated unit cost. Finally, differences in total costs and total QALYs were computed to calculate the ICERs.

Local data were referenced when available. European or American data mostly were utilized in circumstances where there was insufficient local data or when international evidence was determined to be of higher quality and more valid than evidence derived from the Malaysian setting. The model included all targeted populations that were at risk of influenza-related illness, as identified in a published report^37^. Life expectancy for Malaysians above 60 years were obtained from national statistics^38^ and World Data Atlas^39^. The range of input values and relevant sources included for each parameter in the model are summarized in Table 1.

### Analysis

#### Base case analysis

In the cost-utility analysis, the targeted study cohort used for CEA was stratified into two age groups within the vaccinated and unvaccinated study populations. Base case analysis was conducted based on third-party payer and societal perspectives. From a third-party payer perspective, the model only included estimated health costs directly associated with treating, managing, and caring for patients with influenza, which included consultations, hospitalizations, and prescriptions. Indirect costs, specifically productivity loss due to influenza and OTC medications, were included for a societal perspective. The costs and health outcomes were discounted by an annual rate of 3%^40^. The cost-effectiveness threshold of willingness to pay were set at USD 30,984, which was equivalent to three times the GDP per capita in Malaysia as recommended by the World Health Organization^26^.

#### Sensitivity analysis

Deterministic sensitivity analyses (DSA) were conducted to test the effect of single variables on the overall economic conclusions of the model. The tornado diagram was used to visually demonstrate the resulting ICERs when one variable was changed to either the maximum or minimum value within its range. The diagram was used to identify the relative importance of a variable since it could demonstrate if changes to a variable could alter the economic conclusion. A variation of ± 20% was assumed for all parameters except for the discount rate, for which the value was varied from 0 to 5% (Table 1). In an ICER tornado diagram, the importance of each variable on the economic conclusion was positioned from top to bottom. The tails of each bar indicated the maximum and minimum ICER for each variable. The dashed line represented the ICER from the reference case, as a reference for changes in ICERs.

Subsequently, a multivariate analysis was performed with a probabilistic sensitivity analysis (PSA). Instead of changing one parameter value at a time, the PSA changed all variables at once according to their plausible values by random sampling from their distributions. The model was simulated 1000 times with the PSA from the probability distribution of each parameter. All costs and influenza-related outcome rates such as influenza-related GP consultation rate, influenza-related hospitalization rate and influenza-related mortality rate were assigned to a log-normal distribution. Utility data, vaccine coverage rate, percentage of patients consulting a physician, strain circulation and vaccine efficiency followed a beta distribution (Table 1). The cost-effectiveness scatterplot was used to test the stability of the model results. All costs are reported in 2020 United States dollars (USD), converted from Malaysian Ringgits (MYR) using the exchange rate as in 2020 (4.2 MYR = 1 USD).

## Results

### Base case analysis

#### Health-related outcome

The cost-effectiveness model predicted that the use of QIVs in the elderly population in Malaysia would prevent 19,235 cases of influenza that required medical consultation, 47,091 cases that led to a GP visit, 3195 hospitalizations, and 888 deaths. Furthermore, the cost-utility model predicted that the use of QIVs would avoid 79,206 lost workdays. The QIVs would result in 10,048 QALYs gained and 11,160 life years gained. Outcomes results were also reported in age-stratified groups of 64–74 years and ≥ 75 years (Table 2).

**Table 2:** Health outcomes avoided with quadrivalent influenza vaccines for the elderly in Malaysia.

	Age group		
	60–74	75 and above	Total
Non consulting cases	10,727	8508	19,235
General practitioner visits	26,259	20,832	47,091
Influenza cases	36,986	29,340	66,326
Hospitalizations	1779	1416	3195
Deaths	495	393	888
Life years gained	7760	3400	11,160
Quality-adjusted life years gained	6925	3123	10,048
Workdays saved	44,168	35,038	79,206

#### Cost-utility analysis

The use of QIV in an influenza vaccination strategy would save costs on GP consultations, hospitalizations, prescription medicine, OTC medications and productivity losses due to illness and death compared with no vaccination. It would reduce productivity losses by approximately USD 21.6 million and would save USD 4.4 million currently spent on influenza-related hospitalizations (Table 3). From the third-party payer perspective, the incremental cost per QALYs gained between QIV and no vaccination was USD 2,216, which was lower than one per capita GDP of the country (Table 4). Given that the World Health Organization defined a cost effective intervention as being less than three times the national annual GDP per capita, the QIV was highly cost effective^41^.

**Table 3:** Cost saved with the use of QIV for the targeted population in Malaysia (USD).

	Age group		
	60–74	75 and above	Total
General practitioner consultations	185,065	146,811	331,876
Hospitalizations	2,485,683	1,978,584	4,464,267
Prescription	178,750	141,801	320,552
Over-the-counter medication	404,763	321,096	725,860
Productivity losses due to illness	1,754,521	1,391,849	3,146,371
Productivity losses due to death	12,822,785	5,618,409	18,441,194

**Table 4:** Total cost associated with influenza for no-vaccination and QIV from the third-party payer perspective, incremental cost and incremental cost-effectiveness ratio (ICER).

Age groups	Total cost of no vaccination (USD)	Total cost with QIV (USD)	Incremental cost with QIV (USD)	QALYS gained	ICERs (USD)
60–74 years	6,005,011	24,710,588	18,705,577	6925	2701
75 + years	4,806,933	8,365,137	3,558,204	3123	1139
Total	10,811,944	33,075,725	22,263,781	10,048	2216

#### Sensitivity analyses

**Figure 1:**
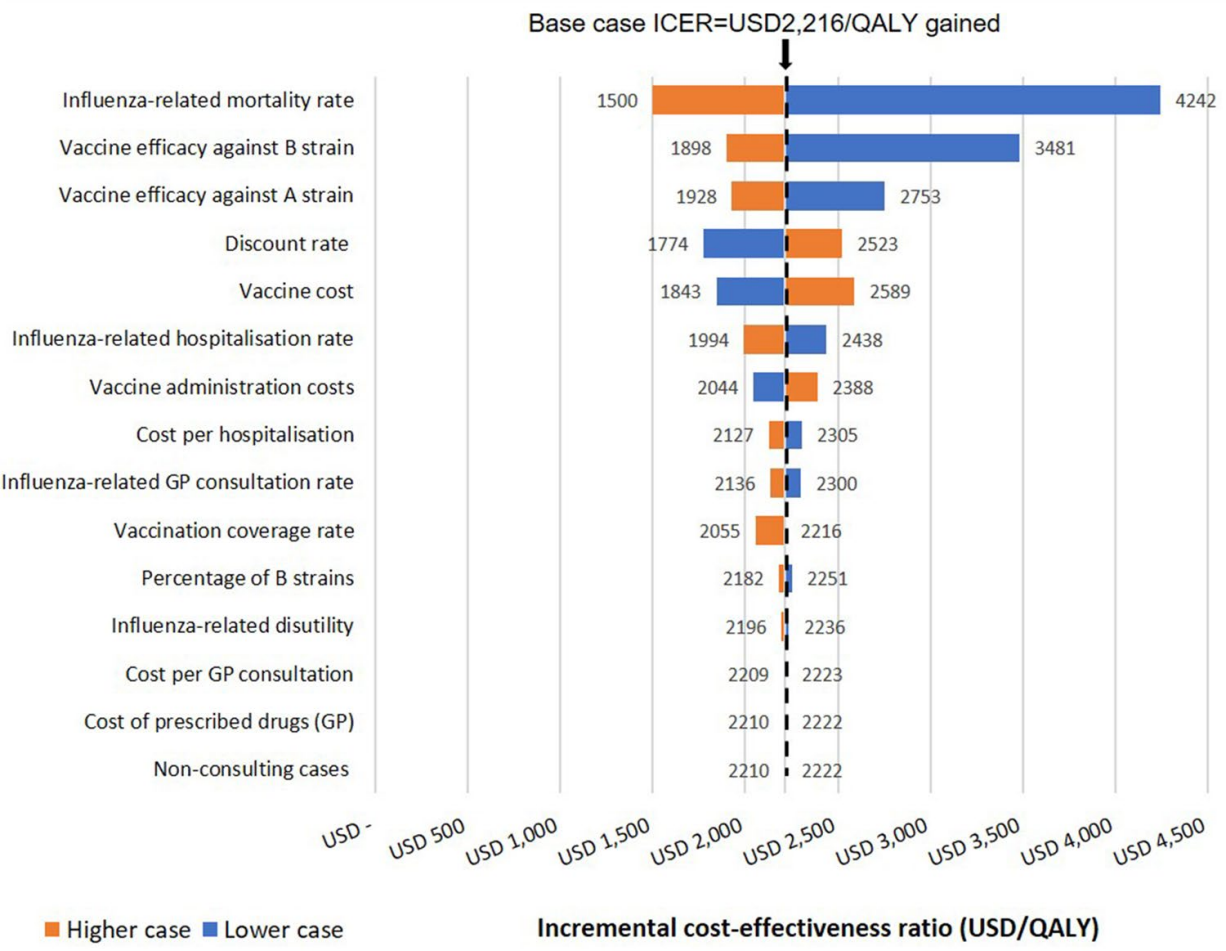
Deterministic sensitivity analysis. *GP* general practitioner.

In this study, we conducted multiple one-way sensitivity analyses to assess the impact of uncertainty in various parameters as listed in Table 1. The results of the analyses demonstrated that this CEA model was most sensitive to the influenza-related mortality rate (from USD 4242 in the low-case scenario to USD 1500 in the high-case scenario) followed by vaccine efficiency against B strains (from USD 3481 in the low-case scenario to USD 1898 in the high-case scenario) and vaccine efficiency against A strains (from USD 2753 in the low-case scenario to USD 1928 in the high-case scenario), as depicted in Fig. 1.

**Figure 2:**
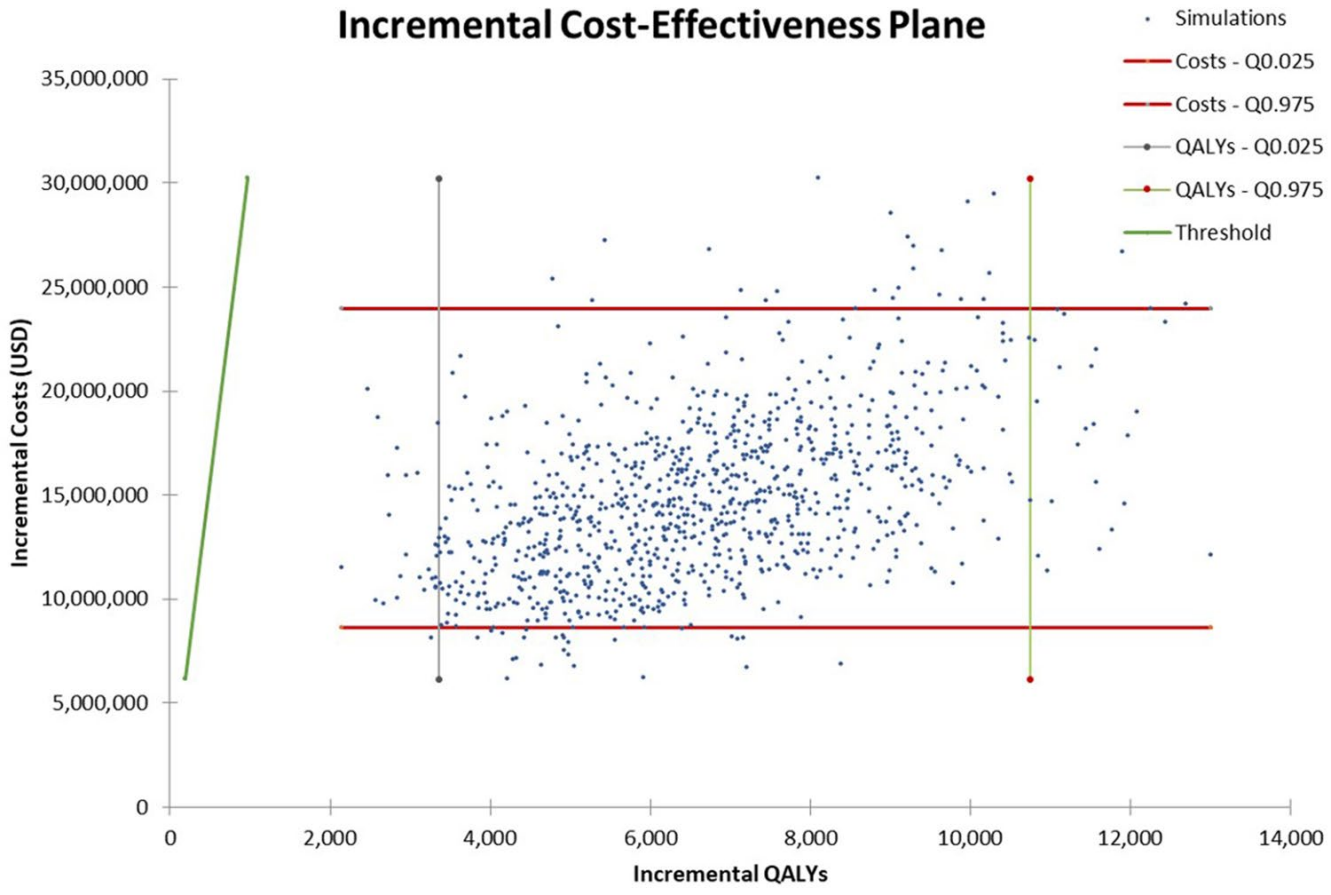
Scatter plot of incremental costs versus incremental QALYs in probabilistic sensitivity analysis (PSA). QALYs quality-adjusted life years.

In probabilistic simulations, where the ICERs from the PSA were plotted onto the cost-effectiveness plane, all the outcomes were located to the right of the threshold lines in quadrant I (i.e. cost-effective). The minimum and maximum ICERs obtained through the PSA were USD 817 and USD 8138, respectively. This finding confirmed that QIV was a cost-effective option in the Malaysian elderly compared with no-vaccination (Fig. 2).

## Discussion

In this study, we conducted a full health economy evaluation analysis with the aim of applying the economic modelling method to evaluate the cost-effectiveness of QIV compared with no-vaccination among Malaysians aged over 60 years. Although high-dose QIV is strongly advised for the elderly^11^, the standard dose was administered in this study due to its availability in Malaysia. The cost-utility model showed that the QIV was highly cost-effective from the third-party payer’s perspective as the ICER estimates was USD 2216, below the threshold of three times national GDP per capita value (USD 30,984). The population aged 60 to 74 years demonstrated the greatest improvements in health outcomes when compared to much older group. The observed result can be attributed to the higher population size within this age category in comparison to individuals aged 75 years and older. Furthermore, the vaccine efficacy is reduced in this population as a result of the changes in their immune system, known as immunosenescence, which can weaken immune responses. This phenomenon may result in a compromised capacity for generating a robust immune response to vaccinations, thereby diminishing their efficacy.

The cost saved was found to be higher among the population aged 60 to 74 years, of which productivity lost due to illness and death dominated the cost saved (USD 14.6 million) followed by hospitalization cost (USD 2.5 million). The cost saved was calculated based on the health outcome avoided by multiplying each case by their respective cost. In this study, hospitalization cost was collected from the casemix database from a teaching hospital in Malaysia. Inpatient cost was estimated at USD1397 per admission among the elderly patients infected with influenza-related disease. The findings of our study revealed a little discrepancy when compared to a prior study, which documented an average cost of USD 8330 for individuals between the ages of 65 and 84 who were admitted to the hospital^9^. Higher costs observed in this age group were attributed to infection-related complications and an extended hospitalization period.

Overall, the adoption of QIV would incur more cost. From the base-case estimates, the use of QIVs would cost USD 22,263,781 more than no vaccination. There were limitations to comparing this finding with existing studies, given that most existing studies compared QIVs with trivalent vaccines (TIV)^35,42^. Nevertheless, when QIVs was compared with the TIV, the cost of QIVs was deemed higher than trivalent vaccines. For instance, a study conducted in Taiwan reported an additional cost of USD 394,000 when switching from trivalent vaccines to QIV^43^. However, this Taiwanese study reported an additional 10,557 QALYs with the utilization of QIVs that yielded an ICER of USD 3015.07. This finding was consistent with the finding of our analysis, which demonstrated that the usage of QIVs would lead to 10,048 QALYs gained and would yield an ICER of USD 2216. Furthermore, the ICER reported by our study was lower than the threshold, which established that QIV was cost-effective. Hence, the adoption of QIVs would be cost-effective and reduce the risk of pre-mature deaths related to influenza, despite showing increments in vaccination costs^35,42–46^.

Based on the guideline on economic evaluation of immunization programs provided by the World Health Organization (WHO), the utilization of static models was deemed suitable for assessing the effectiveness of influenza vaccination within populations that do not significantly contribute to disease transmission. Compared to dynamic models, static models do not account for a herd immunity effect, which can occur particularly in younger populations. However, this should have little effect on our results, as the primary focus of this study was on elderly people. Besides, the vaccine coverage was significantly low among the older people in Malaysia, the indirect impact of herd immunity would be limited.

In this study, we calculated the patient’s and caregiver’s productivity losses based on the duration of hospitalization and outpatient visits from the Casemix database and expert input^2^. According to a current study conducted in Malaysia, 16% of the elderly aged over 65 years are currently employed^27^. Therefore, the estimation of absenteeism due to workdays lost as well as productivity loss due to pre-mature death for the elderly population was included to reflect the societal perspective. However, the estimation for daily productivity was based on GDP per capita owing to the fact that information on the elderly’s salary was not available. From the third-party payer perspective, loss of productivity due to influenza and premature death was not included in the total costs or in the ICER calculations. Nevertheless, incorporating productivity loss costs in the present study would provide a detailed estimation of the indirect costs of QIV vaccination and influenza disease events. This allows policymakers to make informed decisions that not only improve public health outcomes but also contribute to economic well-being and societal development.

The developed model had several potential limitations. First, due to insufficient national data, estimates of influenza hospitalization rates and GP consultation rates were based on published literature from other countries^33^. However, these rates were calculated and adjusted based on mortality rates estimated for Malaysia from a previous study^32^. Thereby, the estimates reflected to the Malaysia socio-economic and appropriate to apply for the Malaysian elderly population. While this may have affected outcome estimates, the sensitivity analysis shows that the effect was minimal in the base case. Second, we adjusted the vaccine efficacy data from previous literature to fit the age groups used in the model. Notably, the vaccine efficacy did have an impact in the study model, as shown in the sensitivity analysis. However, most important finding of the study was that QIVs were effective for preventing death and improve quality of life in the elderly. It was presumed that the influenza vaccination efficacy (IVE) was equivalent for influenza A(H1N1) and A(H3N2); a similar assumption was made by a previous study^35^. In our case, this assumption should not have impacted the evaluation of QIV when compared to no vaccination. Finally, due to limited data availability, the value for QALY lost due to influenza was assumed to be the same as a study done in the UK^30^. This was the most reliable data conducted on the elderly patient and appropriate to apply to the Malaysian population since there was no such data available in Asia and neighboring countries. The value was also comparable with another study conducted in Spain whereby QALY lost in the elderly aged 65 years and above was at 0.0061^47^. The application of disutility value from UK and other high-income countries was also utilized by the Korean and Thailand studies^21,48^. Nevertheless, the multiple-one-way sensitivity analysis also demonstrated this parameter had limited impact on the ICER result. The utility score from EQ 5D has not yet been established for Malaysian population. For this reason, the EQ VAS was used as an alternative to the EQ-5D for measuring health-related utility norms for elderly population in this study. The EQ VAS is a vertical visual analog scale where respondents rate their current health state on a scale from 0 to 100, with 0 representing the worst health imaginable and 100 representing the best health imaginable. This provides a direct measure of the respondent’s overall perception of their health status.

This study is likely to be the first study in Malaysia that has conducted a comprehensive analysis of influenza vaccination strategy in the elderly in terms of cost and health outcomes. The existing guideline for economic evaluation study was established by the Ministry of Health Malaysia, provide a structured framework for conducting economic assessments of healthcare interventions and technologies^49^. This study complied to the guideline to ensure that cost-effectiveness analysis is conducted systematically, transparently, and consistently, enabling policymakers, healthcare professionals, and researchers to make informed decisions about resource allocation on influenza immunization program in the elderly population in Malaysia. Our study conclusively demonstrates that the implementation of an influenza vaccination program in the elderly population is notably cost-effective, aligning with guidelines that deem interventions costing less than one GDP as having a higher probability of being considered for funding^49^. Based on the findings, we put forth recommendations for the Ministry of Health (MOH) to incorporate an influenza immunization program for the elderly into the Malaysia’s National Immunization Program.

## Conclusions

Influenza-related complications are more common in people over the age of 65. Moreover, they were demonstrated to be particularly vulnerable during the COVID-19 pandemic, thus underscoring the need to increase immunogenicity and avoid premature death in this high-risk group with effective influenza vaccines. This economic model suggested that although QIV would incur higher costs, it led to the highest QALYs gained. Our study provides compelling evidence of the benefits of influenza vaccination program, including its potential to reduce disease burden, improve public health, and save healthcare costs. It is apparent that influenza vaccination is the dominant strategy when considering all health outcome consequences in this assessment. Hence, compared to no-vaccination, QIV would reduce the burden of managing influenzas especially among the elderly.

## Data Availability

The datasets generated during and/or analyzed during the current study are available from the corresponding authors on reasonable request.

## References

[1] Thompson MG (2018). Influenza vaccine effectiveness in preventing influenza-associated intensive care admissions and attenuating severe disease among adults in New Zealand 2012–2015. Vaccine.

[2] Mad Tahir NS (2022). Clinical pathway for influenza in the elderly: A comprehensive management protocol of Malaysia. Asia Pac. J. Heal. Manag..

[3] World Health Organization. Influenza (seasonal). Fact sheet. https://www.who.int/en/news-room/fact-sheets/detail/influenza-(seasonal) (2018).

[4] Sam JICC (2015). The burden of human influenza in Malaysia. Med. J. Malays..

[5] Saat Z (2010). Seasonal influenza virus strains circulating in Malaysia from 2005 to 2009. Southeast Asian J. Trop. Med. Public Health.

[6] Sam IC (2019). Seasonal influenza activity based on laboratory surveillance in Malaysia, 2011–2016. J. Med. Virol..

[7] World Health Organization (2012). Vaccines against influenza WHO position paper-November 2012. Wkly. Epidemiol. Rec..

[8] Arriola C (2017). Influenza vaccination modifies disease severity among community-dwelling adults hospitalized with influenza. Clin. Infect. Dis..

[9] Putri WCWS, Muscatello DJ, Stockwell MS, Newall AT (2018). Economic burden of seasonal influenza in the United States. Vaccine.

[10] Tokars JI, Rolfes MA, Foppa IM, Reed C (2016). An evaluation and update of methods for estimating the number of influenza cases averted by vaccination in the United States. Physiol. Behav..

[11] Centers for disease control and prevention. Fluzone high-dose seasonal influenza vaccine (CDC). https://www.cdc.gov/flu/prevent/qa_fluzone.htm?web=1&wdLOR=c9B48F9AB-3E41-4A81-B3DD-8BEBC075D8E4 (2021).

[12] Tan MP (2022). A Malaysian consensus recommendation for the prevention of influenza in older persons. BMC Infect. Dis..

[13] Shaharudin, A. Influenza vaccination for the elderly and economic evaluation. https://www.moh.gov.my/index.php/database_stores/attach_download/347/346 (2019).

[14] Wong PL (2020). The effects of age on clinical characteristics, hospitalization and mortality of patients with influenza-related illness at a tertiary care centre in Malaysia. Influenza Other Respir. Viruses.

[15] Muhammad Azami NA (2023). Hepatitis B and influenza vaccination coverage in healthcare workers, the elderly, and patients with diabetes in Malaysia. Hum. Vaccines Immunother..

[16] Chit A, Roiz J, Aballea S (2015). An assessment of the expected cost-effectiveness of quadrivalent influenza vaccines in Ontario, Canada using a static model. PLoS One.

[17] Barbieri M, Capri S, Waure CDE, Boccalini S, Panatto D (2017). Age- and risk-related appropriateness of the use of available influenza vaccines in the Italian elderly population is advantageous: Results from a budget impact analysis. J. Prev. Med. Hyg..

[18] Nguyen VH, Vizzotti C, Uruena A, Giglio N, Magneres C, Richmond H (2020). Cost-effectiveness of introducing an MF59-adjuvanted trivalent influenza vaccine for older adults in Argentina. Vaccine.

[19] Van Bellinghen LA, Meier G, Van Vlaenderen I (2014). The potential cost-effectiveness of quadrivalent versus trivalent influenza vaccine in elderly people and clinical risk groups in the UK: A lifetime multi-cohort model. PLoS One.

[20] Yun JW (2019). Cost-effectiveness of influenza vaccine strategies for the elderly in South Korea. PLoS One.

[21] Choi EJ, Park JH, Chun BC (2020). Cost effectiveness of trivalent and quadrivalent influenza vaccines in 50- to 64-year-old adults in Korea. Vaccine.

[22] Rashid SAZA, Nur AM, Wan Puteh SE, Aljunid SM (2017). Incidence of clinical coding errors and implications on Casemix reimbursement in a teaching hospital in Malaysia. Malays. J. Public Health Med..

[23] Aljunid SM, Al Bashir L, Ismail AB, Aizuddin AN, Rashid SAZA, Nur AM (2022). Economic impact of switching from partially combined vaccine “Pentaxim® and hepatitis B” to fully combined vaccine “Hexaxim®” in the Malaysian National Immunization Program. BMC Health Serv. Res..

[24] Abdul Aziz AF, Mohd Nordin NA, Muhd Nur A, Sulong S, Aljunid SM (2020). The integrated care pathway for managing post stroke patients (iCaPPS©) in public primary care health centres in Malaysia: Impact on quality adjusted life years (QALYs) and cost effectiveness analysis. BMC Geriatr..

[25] Yuasa A, Yonemoto N, LoPresti M, Ikeda S (2021). Use of productivity loss/gain in cost-effectiveness analyses for drugs: A systematic review. Pharmacoeconomics.

[26] Department of Statistics Malaysia. Gross domestic product (GDP) by state 2020. https://www.dosm.gov.my/v1/index.php?r=column/ctwo&menu_id=TE5CRUZCblh4ZTZMODZIbmk2aWRRQT09 (2021).

[27] Alkhodary AA, Aljunid SM, Ismail A, Nur AM, Shahar S (2022). Health care utilization and out-of-pocket payments among elderly with cognitive frailty in Malaysia. Int. J. Environ. Res. Public Health.

[28] Department of Statistics Malaysia. Labour force, Malaysia: April 2020. Department of Statistics Malaysia (2020).

[29] Shafie AA, Vasan A, Lim CJ, Luo N (2018). Psychometric performance assessment of Malay and Malaysian English version of EQ-5D-5L in the Malaysian population. Qual. Life Res..

[30] Camacho A, Eames K, Adler A, Funk S, Edmunds J (2013). Estimation of the quality of life effect of seasonal influenza infection in the UK with the internet-based Flusurvey cohort: An observational cohort study. Lancet.

[31] Simmerman JM (2006). The cost of influenza in Thailand. Vaccine.

[32] Luliano DA (2018). Estimates of global seasonal influenza-associated respiratory mortality: A modelling study. Lancet.

[33] Molinari NAM (2007). The annual impact of seasonal influenza in the US: Measuring disease burden and costs. Vaccine.

[34] FluNet. Influenza laboratory surveillance information. Global Influenza surveillance and response system (GISRS). https://www.who.int/tools/flunet (2021).

[35] Mennini FS, Bini C, Marcellusi A, Rinaldi A, Franco E (2018). Cost-effectiveness of switching from trivalent to quadrivalent inactivated influenza vaccines for the at-risk population in Italy. Hum. Vaccines Immunother..

[36] Clements KM, Meier G, McGarry LJ, Pruttivarasin N, Misurski DA (2014). Cost-effectiveness analysis of universal influenza vaccination with quadrivalent inactivated vaccine in the United States. Hum. Vaccines Immunother..

[37] Department of Statistics Malaysia. Current Population Estimates, Malaysia, 2020. https://www.dosm.gov.my/v1/index.php?r=column/cthemeByCat&cat=155&bul_id=OVByWjg5YkQ3MWFZRTN5bDJiaEVhZz09&menu_id=L0pheU43NWJwRWVSZklWdzQ4TlhUUT09# (2020).

[38] Department of Statistics Malaysia. Abridged Life Tables, Malaysia, 2019–2021. https://www.dosm.gov.my/v1/index.php?r=column/pdfPrev&id=R0VPdE1mNEdRQms2S0M4M1ZsSlVEdz09 (2021).

[39] Knoema World Atlas Data. Malaysia Life expectancy at age 60 years, 1950–2021. https://knoema.com/atlas/Malaysia/topics/Demographics/Age/Life-expectancy-at-age-60-years (2021).

[40] Drummond, M.F., Sculpher, M.J., Claxton, K., Stoddart, G.L. & Torrance, G.W. Methods for Economic Evaluation of Health Care Programmes. Oxford: Oxford Medical Publications. https://www.researchgate.net/publication/227467531_Methods_for_The_Economic_Evaluation_of_Health_Care_Programmes (2002).

[41] Marseille E, Larson B, Kazi DS, Kahn JG, Rosen S (2015). Thresholds for the cost–effectiveness of interventions: Alternative approaches. Bull. World Health Organ..

[42] Van Bellinghen LA, Marijam A, de Araujo GTB, Gomez J, Van Vlaenderen I (2018). Cost-utility of quadrivalent versus trivalent influenza vaccine in Brazil—Comparison of outcomes from different static model types. Braz. J. Infect. Dis..

[43] Yang MC, Tan ECH, Su JJ (2017). Cost-effectiveness analysis of quadrivalent versus trivalent influenza vaccine in Taiwan: A lifetime multi-cohort model. Hum. Vaccines Immunother..

[44] Chit A, Roiz J, Briquet B, Greenberg DP (2015). Expected cost effectiveness of high-dose trivalent influenza vaccine in US seniors. Vaccine.

[45] de Boer PT (2018). The cost-effectiveness of trivalent and quadrivalent influenza vaccination in communities in South Africa, Vietnam and Australia. Vaccine.

[46] García A, de Lejarazu RO, Reina J, Callejo D, Cuervo J, Larragueta RM (2016). Cost–effectiveness analysis of quadrivalent influenza vaccine in Spain. Hum. Vaccines Immunother..

[47] Redondo E (2021). Cost-utility analysis of influenza vaccination in a population aged 65 years or older in Spain with a high-dose vaccine versus an adjuvanted vaccine. Vaccine.

[48] Suphanchaimat R (2020). Cost effectiveness and budget impact analyses of influenza vaccination for prisoners in thailand: An application of system dynamic modelling. Int. J. Environ. Res. Public Health.

[49] Ministry of Health Malaysia. *Pharmacoeconomic guideline for Malaysia: Second edition*. https://www.pharmacy.gov.my/v2/sites/default/files/document-upload/pharmacoeconomic-guidelines-malaysia-malaysia-second-edition-2019-final-page-adjustment.pdf (2019).

